# Molecular characterization of *SPL* gene family during flower morphogenesis and regulation in blueberry

**DOI:** 10.1186/s12870-023-04044-x

**Published:** 2023-01-18

**Authors:** Xin Feng, Bingjie Zhou, Xinliang Wu, Huiling Wu, Suilin Zhang, Ying Jiang, Yaping Wang, Yaqian Zhang, Man Cao, Baoshi Guo, Shuchai Su, Zhixia Hou

**Affiliations:** Key Laboratory for Silviculture and Conservation of Ministry of Education, Research and Development Center of Blueberry, Beijing Forestry University, Beijing, 100083 China

**Keywords:** *Vaccinium corymbosum L*, *SPL* gene, Floral induction, Phylogenetic analysis, Expression profile

## Abstract

The *SPL* gene is a plant-specific transcription factor involved in the regulation of plant growth and development, which have been identified in woody plants. The process of floral bud differentiation affects the timing of flowering and fruit set and regulates plant growth, however, the mechanism of regulation of flower development by *SPL* genes is less studied. In this study, 56 *VcSPL* genes were identified in the tetraploid blueberry. The *VcSPL* gene family was classified into six subfamilies, and analysis of cis-elements showed that *VcSPL* genes were regulated by light, phytohormones (abscisic acid, MeJA), and low temperature. In the evolutionary analysis, segmental replication may play an important role in *VcSPL* gene amplification. Interestingly, we also studied diploid blueberry (Bilberry), in which 24 *SPL* genes were identified, and 36 homologous pairs were found, suggesting a high degree of convergence in the syntenic relationship between blueberry (*Vaccinium corymbosum* L) and bilberry (*Vaccinium darrowii*). Based on the expression profile, *VcSPL* genes were expressed at high levels in flowers, shoots, and roots, indicating a diversity of gene functions. Then we selected 20 differentially-expressed *SPL* genes to further investigate the role of *VcSPL* in floral induction and initiation. It showed that the genes *VcSPL40*, *VcSPL35*, *VcSPL45*, and *VcSPL53* may play a crucial role in the blueberry floral transition phase (from vegetative growth to flower initiation). These results provided important information for understanding and exploring the role of *VcSPLs* in flower morphogenesis and plant growth.

**Supplementary Information:**

The online version contains supplementary material available at 10.1186/s12870-023-04044-x.

## Introduction

Plant transcription factors are DNA-binding proteins that regulate gene expression at the transcriptional level [1] and are essential for plant growth and development, participating in the regulation of several plants physiological and biochemical processes [2]. The SQUAMOSA-promoter binding protein-like (*SPL/SBP*) gene was first identified in Antirrhinum majus as a transcription factor regulating early flower development [3]. The gene family was named after a conserved DNA-binding domain of 78–80 bp amino acid residues and was characterized by two DNA-binding domains (zinc finger structures) and a C-terminal NLS nuclear localization signal that overlapped with the zinc finger structure [4]. The *SPL* gene family has been identified in several cultivated economic trees, such as litchi [5], kiwifruit [6], chestnut [7], citrus [8], sweet orange [9], apple [10], grape [11], jujube [12], walnut [13], pecan [14]. In these plants, *SPL* genes were involved in a wide range of plant growth processes, including vegetative growth, flower development, fruit development and ripening, and abiotic stresses. The major *SPL* gene family had important roles in plant flower development, including vegetative to reproductive growth [15–18], anther development [19], GA flower regulatory pathways [16], and flower morphogenesis [20]. In Arabidopsis, *AtSPL3* and its homologs *SPL4* and *SPL5* were highly expressed in flowering shoots and involved in flowering [21, 22], and *AtSPL3* was directly regulated by the *SOC1* gene [23]. Transcript levels of *AtSPL4* were decreased in the double loss-of-function mutant of *SOC1* and *FUL* [15]. Moreover, *AtSPL3*, *AtSPL4*, and *AtSPL5* are involved in the regulated floral transition, which requires synergy with the FT-FD module to promote floral meristem transformation under long sunlight [16].On the other hand, *AtSPL9/AtSPL15* and *AtSPL13* were involved in juvenile to adult growth phase transition [15, 16], with *AtSPL9* being associated with leaf growth rate and final size [17]. The miRNA-*SPL* regulatory network was involved in floral transition and development [20, 24]. miR156 played a key role in vegetative phase change and shoot development. And overexpression of miR156 in Arabidopsis led to a prolonged juvenile to adult phase [20]. In *Arabidopsis thaliana*, under short-day conditions, the *SPL* gene targeting miR156 had an important role in floral transition by activating the MADS-box gene at the shoot apical [20]. Under long-day conditions, in addition to a similar role at the shoot apical, the interaction between DELLA and *SPL* interfered with *SPL* transcriptional activity, thereby delaying floral transformation under long daylight conditions by inactivating miR172 in leaves and the MADS-box gene at the shoot apex [20]. Meanwhile, *AtSPL9* /*AtSPL15* interacted with the GA signalling protein DELLA, which was involved in the GA-mediated flowering pathway [16]. In addition *AtSPL2*, *AtSPL10*, *AtSPL11*, and *AtSPL13* were involved in the regulation of the vegetative-reproductive transition [18], and *AtSPL8* played an important role during sporophyte development [19]. In addition to dicotyledons, *SPL* genes also played an important role in monocotyledons. In rice, *OsSPL10* was preferentially expressed in young panicle and stem, and negatively controls salt tolerance but positively controls trichome formation [25], the *SPL14* mutant increased inflorescence branch number and grain weight as well as increasing rod strength [26, 27], and *OsSPL2*, *OsSPL17* and *OsSPL18* regulated tiller number and plant height [28].

In other woody plants, *SPL* genes also play an important role in flower development. GA might indirectly influence the expression of its target genes *CmSPL6/CmSPL9/CmSPL16* through miR156, thereby playing an important role in chestnut flower bud development [7]. Grape *VpSBP11* worked as a transcription factor during the transition from nutritional to reproductive stages, and overexpression in Arabidopsis activated the expression of *FUL, AP1*, and *LFY* genes, thus showing early flowering [29]. Overexpression of *CclSBP6* and *CclSBP7* in citrus could alter flowering time in *Arabidopsis* [8], and similarly, overexpression of *EjSPL3*, *EjSPL4*, *EjSPL5,* and *EjSPL9* in litchi could significantly promote flowering and up-regulated the expression of downstream flowering genes [30]. In addition, the function of *SPL* in floral induction had been reported in walnut (*Juglans regia*) [13] and pecan (*Carya illinoinensis*) [14]. Flower development is an important work in plant growth and development, in which *SPL* is involved as a plant-specific transcription factor, so it is important to study the *SPL* gene family and use its function to regulate the flower development process in plants.

Blueberry is a photoperiod-sensitive berry shrub with short days to promote reproductive growth [31, 32]. The flower bud development process of blueberries is closely related to the flowering and fruiting time, which affects the marketing time of blueberries [33, 34]. At present, greenhouse cultivation has become the important method of blueberry in northern China, which can ensure safe overwintering and earlier fruit ripening and market time [35]. However, compared with open-field blueberry cultivation, the characteristics of the annual growth cycle of blueberries in the greenhouse were significantly different, especially in flower bud differentiation. To better study the process of flower bud development in blueberries, we conducted a genome-wide analysis of the *SPL* gene family in blueberries using genomics [36], including identification of the tetraploid blueberry gene families, phylogeny, chromosomal localization, evolutionary history, and the temporal and spatial expression characteristics during flower development in blueberry. Our study establishes a basis for investigating the regulatory role of *SPL* genes in the blueberry floral induction and initiation processes, which will contribute to the understanding of blueberry plant growth and flower and fruit development.

## Material and methods

### Identification of VcSPLs gene family members

Blueberry genomic data were obtained from (GIGA) ^n^DB (http://gigadb.org/dataset/100537/). HMM and Blast searches were used to predict blueberry *SPL* genes [37, 38]. A total of sixteen *Arabidopsis thaliana* SPL protein sequences [39] were downloaded from NCBI as queries for BLASTP searches (e-value ≤ 1 × 10^−5^). In addition, the SPL DNA-binding domain (PF03110) was obtained from the Pfam database [40], and the HMMER toolkit was adopted to find putative *SPL* genes. The target genes obtained by these two methods were subjected to identification of the *SPL* structural integrity using the SMART program (http://smart.embl-heidelberg.de/). And the target protein properties, including isoelectric point (P), molecular weight (MW), number of amino acids (aa), lipophilicity, and hydrophobicity, were analyzed by the online analysis tool ProtParam (https://web.expasy.org/protparam/). Subcellular localization analysis was performed by the online analysis tool MBC (http://cello.life.nctu.edu.tw/).

### Phylogenetic analysis of VcSPLs

To classify the evolutionary relationships of the *VcSPLs* gene family, the SPL protein sequences of *Arabidopsis thaliana* [39], rice (*Oryza sativa*) [41], tobacco (*Nicotiana tabacum L.*) [42], grape (*Vitis vinifera*) [11] and diploid blueberry (*Vaccinium darrowii*) [43] were aligned using the CluastW algorithm and MEGA11 software [44], where diploid bilberry *SPL* family members were identified in the same way to tetraploid blueberry, with gene names and location information in the Supplementary Table 2. Phylogenetic analysis based on amino acid sequence alignment was performed using the neighbor-joining (NJ) method with Bootstrap tests on 1000 resamples, and for more reliable phylogenetic analysis, maximum likelihood (ML) was also considered [45]. The final phylogenetic relationships were based on the results obtained from the two methods and visualized by EvolView software [46].

### Conserved motifs and gene structure analysis of VcSPLs

Multiple sequence alignment of *VcSPLs* protein sequences was performed using Multiple Sequence Comparison by Jalview Version2 [47]. The motifs of blueberry *SPL* proteins were predicted using an online toolkit of MEME (https://meme-suite.org/meme/tools/meme). The conserved structural domains of blueberry SPL proteins were analyzed by the online toolkit of NCBI CD-Search (https://www.ncbi.nlm.nih.gov/Struct-ure/cdd/wrpsb.cgi). Extraction of target gene sequences from blueberry and visual analysis was performed with TBtools software [48].

### Chromosomal location and duplication events among VcSPL genes

All 56 *VcSPLs* genes were located on the corresponding chromosomes using the blueberry genome information [36]. Based on the genome annotation information, gene densities were extracted by the TBtools [48]. The potential gene duplication events were used as an input file for Multiple Collinearity Scan Toolkit X [49]. The synteny relationship of the orthologous *SPL* genes between bilberry and blueberry, rice, and Arabidopsis were identified using the Synteny visualization plugin embedded in TBtools [48].

### Expression pattern of the VcSPLs genes and analysis of the cis-acting element of the VcSPLs promoter

The fragments per kilobase million (FPKM) values of *Vaccinium corymbosum* L. RNA sequencing data (Bioproject ID#PRJNA494180) were utilized for the gene expression analysis of various tissues [50].

The 2000 bp upstream of the coding regions of the *VcSPL* genes were extracted from the blueberry genome data using by TBtools software. And PlantCARE10 was used to search for putative cis-acting elements (http://bioinformatics.psb.ugent.be/webtools/plantcare/html/).

### Plant material and quantitative RT-PCR analysis

**Fig. 1:**
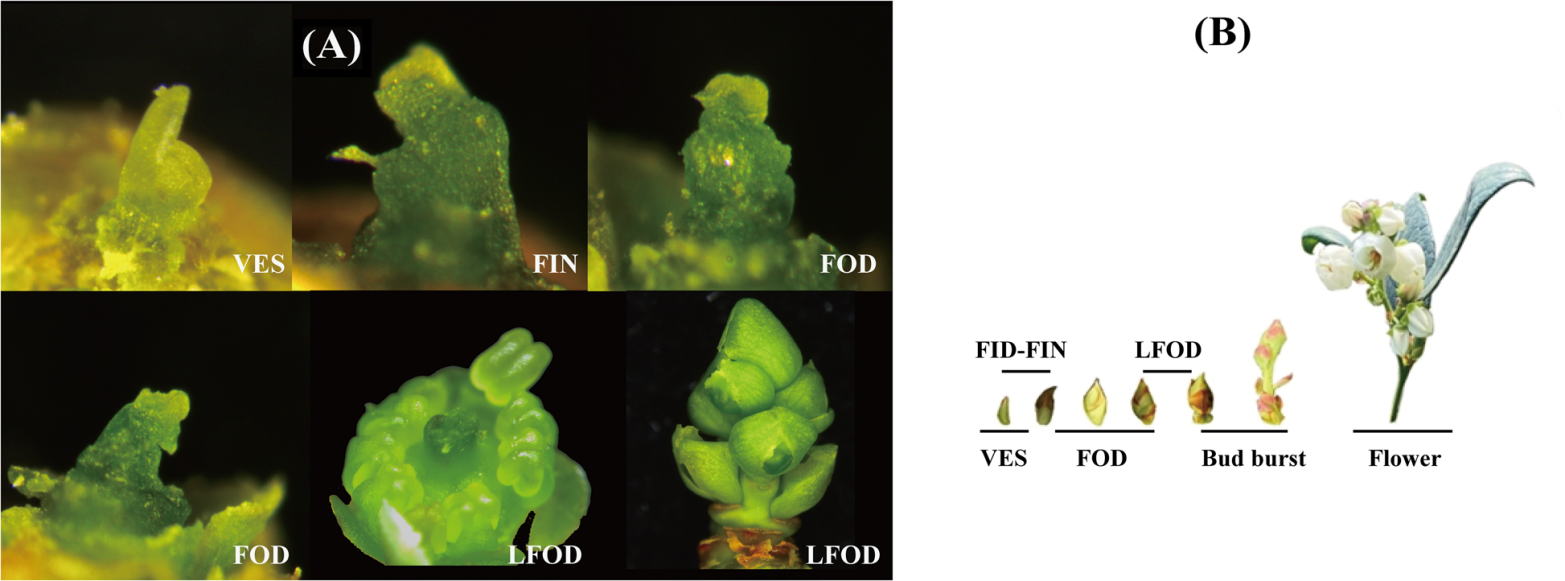
**A-B** The morphology of blueberries at different stages of bud differentiation, stages of the vegetative stage (VES), floral induction stage (FID); floral initiation stage (FIN); floral organ primordium differentiation (FOD); Late floral organ development (LFOD)

The northern highbush blueberry (*Vaccinium corymbosum* L.) plant was used as material, which was collected in the Blueberry Cultivation Garden, Miyun District, Beijing, China (N40°23 “15.86”, N116°47 “2.36”). The four different tissues (shoots, leaves, flower buds, and leaf buds) of blueberries were collected at the time of the bud burst. Genes from each subgroup of the *VcSPL* gene family (derived from evolutionary branches) were selected for differential expression analysis in different tissues. Secondly, to further investigate the role of *VcSPL* in floral induction and initiation, we selected genes that were highly expressed in floral buds, flower at anthesis, and flower post-fertilization for RT-qPCR using samples collected at different stages. A total of five periods in the process of blueberry bud differentiation were selected and the characteristics of the different periods were described in detail (Fig. 1). According to the anatomical characteristics of bud differentiation, the five periods of blueberry bud differentiation were characterized as follows: the vegetative growth stage (VES) showed a narrow meristem. The floral induction stage (FID) appeared in the loosed bud scales and increased the hemispherical of the apical meristem. During the floral initiation stage (FIN), the inflorescence primordium occurred. At the floral organ primordium development stage (FOD), the primordia of the sepals, petals, stamens, and pistil floral have appeared gradually. At the late stage of floral organ development (LOFD), the locules of the ovary developed further, the protruding tips coalesced and grew into the style and stigma, the corolla always coalesced at the tip, the calyx was less than half the length of the corolla, and the complete flower morphology had been presented (Fig. 1).

The total RNA isolation and cDNA synthesis were according to our previous report [51]. Oligo 7 software was used for the design-specific primers, and related primers were shown in Supplementary Table 7. The quantitative kit was Taq Pro Universal SYBR qPCR Master Mix (Vazyme, Nanjing, China). *Actin* and *UBC28* were used as the internal reference gene [52], and three replicates were performed for each sample. The relative gene expression values of the *VcSPL*s gene family were calculated using the 2^−∆∆Ct^ method.

## Results

### Identification of VcSPLs gene family members

To identify SPL-encoding genes in blueberry, we initially performed a local BLAST library build with 16 *SPL* genes from *Arabidopsis thaliana* for comparison, followed by a further screening in combination with PFAM scan analysis to remove redundant sequences and then renaming them according to their position on the chromosome, finally identified a total of 56 *VcSPL* genes (Supplementary Table 1). The previous study indicated that 32 blueberry *SBP/SPL* gene families had been identified and screened, including two alternative *SPL* alternative splicing [53]. However, the previous identification of *SPL* encoding genes was based on a de novo assembly of the transcriptome, which was supplemented by further identification at the level of the whole blueberry genome in this experiment, with 24 new *SPL* gene families identified, and the relationship between the two *SPL* identifications corresponds in Supplementary Table 3. Two alternative splicings were not identified in the whole genome data, and in the previous study, two pairs of genes (*VcSBP14a* and *VcSBP14aAS*, *VcSBP14c*, and *VcSBP14cAS*) could be derived from different RNA splicing of the same genes [53]. In this study, *VcSBP14aAS* and *VcSBP14cAS* were not identified, while *VcSBP14a* and *VcSBP14c* corresponded to *VcSPL43* and *VcSPL30*, respectively, in the present study. This may be due to the different blueberry varieties and tissues sequenced.

The basic information of the *VcSPL* family members, such as amino acid length, molecular weight, theoretical isoelectric point, and subcellular localization, were analyzed by ProtParam. The results (Supplementary Table 1), showed that 56 gene sequences were located on 32 chromosomes, with gene lengths ranging from 1968 bp (Vc*SPL*26) to 9387 bp (Vc*SPL*11). The length distribution of the encoded amino acids ranged from 126aa (Vc*SPL14*) to 1072aa (Vc*SPL43*), and the molecular weight was from 14.04 kDa (Vc*SPL*14) to 117.84 kDa (Vc*SPL*43), and the aliphatic coefficients ranged from 31.90 (*VcSPL38*) to 84.12 (*VcSPL15*). The average hydrophilic coefficients were all less than zero, suggesting that most members of the *VcSPL* transcription factor family were rich in aliphatic amino acids. All members were localized in the nucleus, except for Vc*SPL*36, which was localized in both the cytoplasm and the nucleus.

### Phylogenetic analysis of VcSPLs

**Fig. 2:**
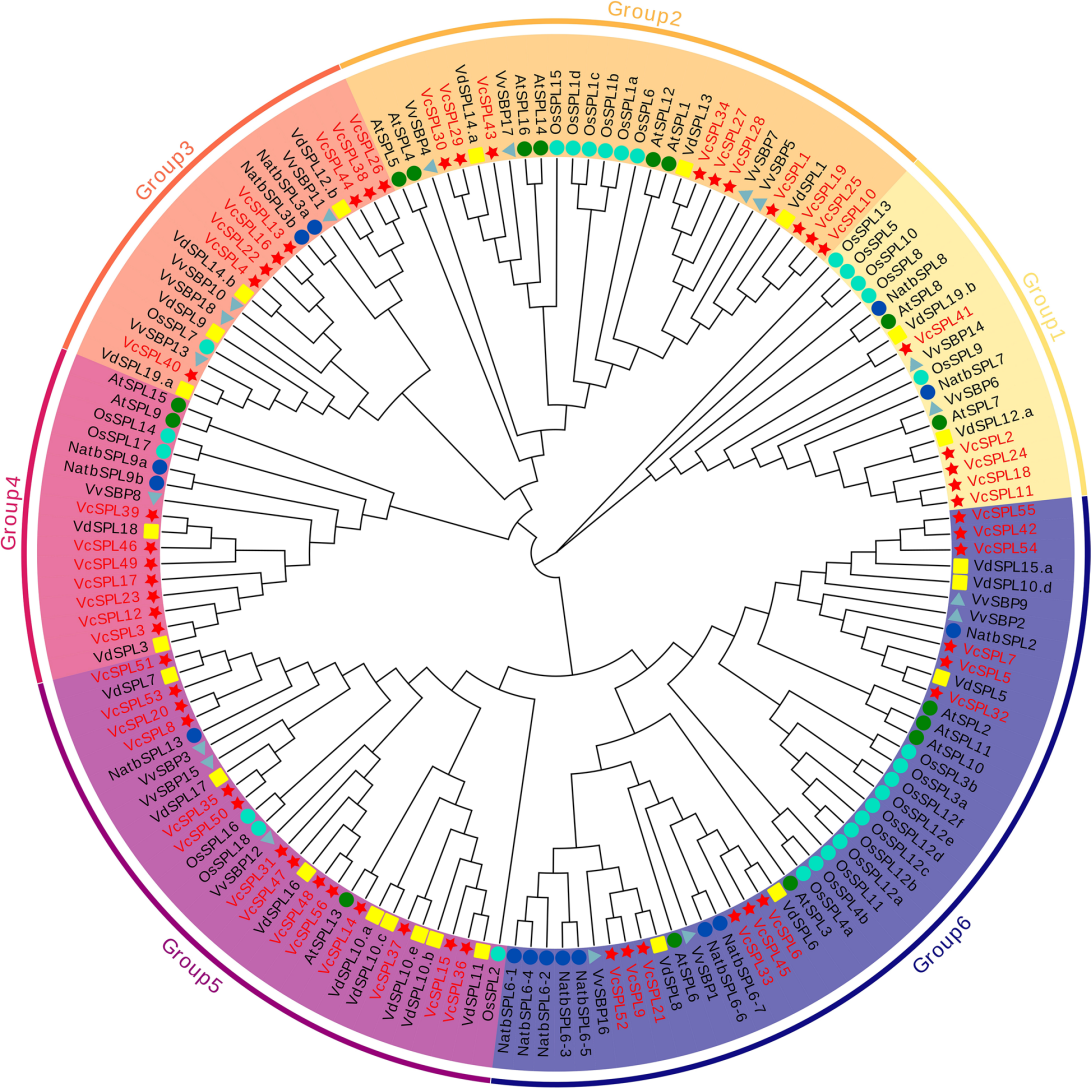
Phylogenetic tree of blueberry (*Vaccinium corymbosum*), *Arabidopsis thaliana*, tobacco (*Nicotiana tabacum*), rice (*Oryza sativa*), grape (Vitis vinifera), and bilberry (*Vaccinium darrowii*) *SPL* proteins. Clades with different colors represent diverse subgroups. They were divided into 6 groups according to the evolutionary tree and were represented by different colours. The different icons represent the *SPL* genes of different species, with red pentagrams representing blueberry, green circles representing Arabidopsis, dark blue circles representing tobacco, cyan circles representing rice, light blue triangles representing grape, and yellow squares representing bilberry

**Fig. 3:**
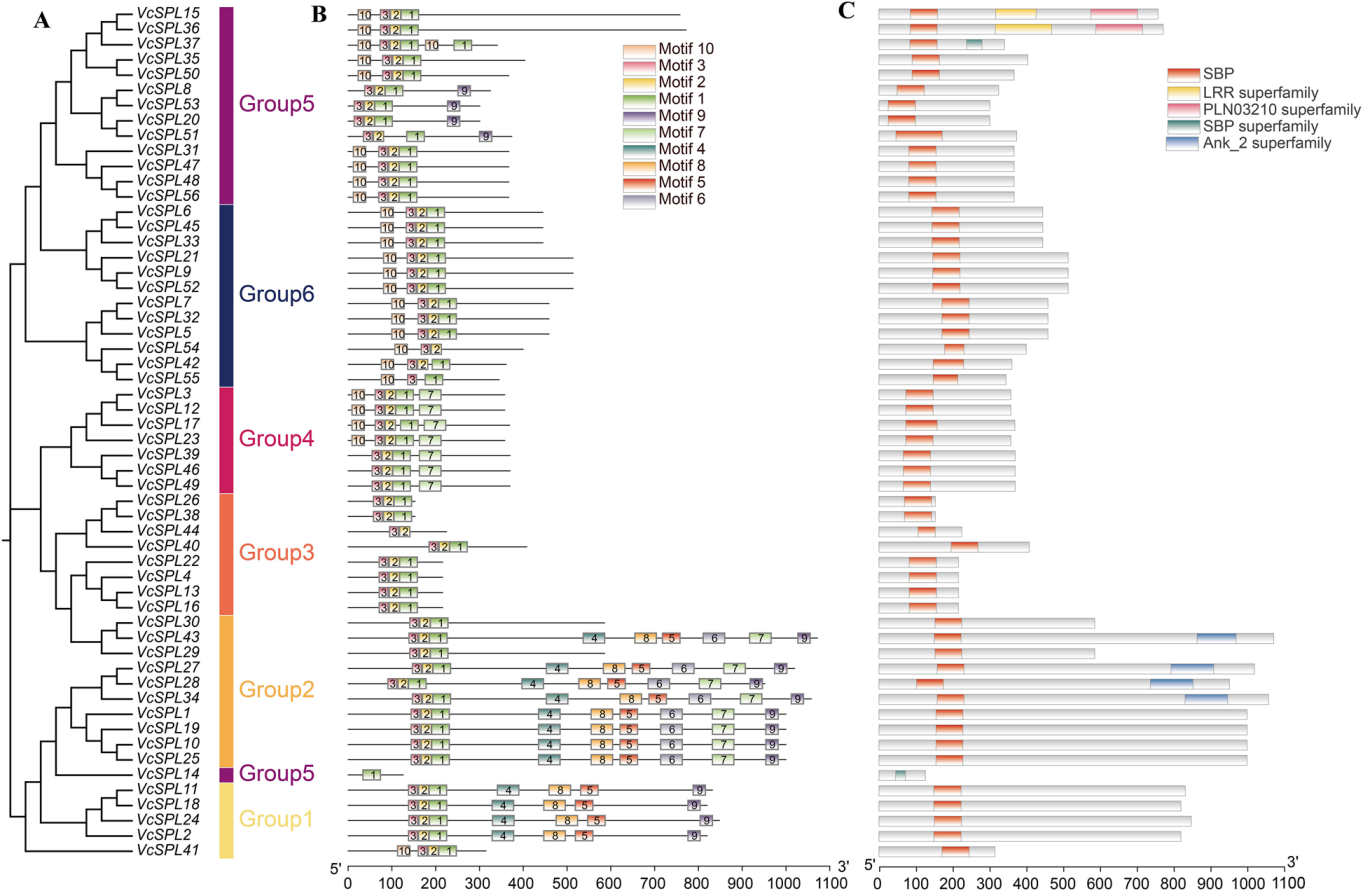
Phylogenetic relationships and protein structure of blueberry *VcSPL*. **A** Neighbor-joining trees constructed for *VcSPL*. **B** Protein motif. Schematic diagram of the conserved motifs of *SPL/SBP* proteins in blueberry, which were elucidated using MEME. Each motif is represented by a number in the coloured box. **C** Conserved domains. Different domains were represented in different colours

To investigate the evolutionary relationships of blueberries, we performed the identification of bilberry *SPL* family members based on recently published diploid blueberry(bilberry) data [43], identifying a total of 24, with gene names and positional information in Supplementary Table 2, and then constructed phylogenetic trees for the amino acid sequences of Arabidopsis, tobacco, rice, and bilberry. The 56 *SPL* structural domains were divided into six subclasses based on topological relationships (Fig. 2). Each group contained at least one blueberry *VcSPL* gene, and Group1 contained the fewest number of *VcSPL*, only five. From the root of the evolutionary tree, the members of the G1 subclass clustered alone among multiple species to form an evolutionary branch, suggesting that it may have a large degree of differentiation among species and that its functions may differ in different plants. In Group2, *VcSPL29/30/43* and *VcSPL10/19/25/27/28/34* can be subdivided into G2a and G2b based on evolutionary branches. The number of Vc*SPL*s in Group5 was at most 14, which can be further divided into two subclasses based on gene structure and conserved motif characteristics (Fig. 3B), namely *VcSPL8*, *VcSPL20*, *VcSPL51*, and *VcSPL53* belong to subclass G5a, while the other members belong to subclass G5b. The other subclasses that have been divided contain similar gene structures and conserved motif features, suggesting that they remained highly conserved during evolution and that the function of these genes was potentially extremely important for the plant body. At the same time, we could see that at the end of the branches, blueberry *SPLs* showed a high degree of clustering with that of bilberry, indicating a high level of homology between the two species, with later differentiation between subspecies, presumably due to the effects of domestication.

### Conserved motifs and gene structure analysis of VcSPLs

Multiple sequence alignment (Fig. S1) of the 56 *VcSPLs* showed that all genes, except Vc*SPL*14, contain essentially two zinc finger structural domains and a nuclear localization signal, and the presence of conserved structural domains suggested that *SPL* family genes might have similar functions that were functionally conserved between species. The phylogenetic tree was built for the *VcSPL* gene family (Fig. 3A), and the classification pattern was consistent with Fig. 2 except for *VcSPL14*, which may be caused by the short amino acid sequence of *VcSPL14* and the lack of a complete zinc-finger structure. In addition, the motifs on each gene were scanned (Fig. 3B), and sequence information of each motif was provided in Fig. S2. The number of motifs ranged from 1–9. Motif1, motif2, and motif3 together formed the complete SPL/SBP conserved structural domain, with motif1 encoding the C2HC zinc finger structure and NLS nuclear localization signal, motif2 and motif3 together encoding the C3H structural domain. However, *VcSPL14* was unable to form a complete C3H zinc finger structural domain because it contained only one motif1 and lacked motif2 and motif3, *VcSPL44* and *VcSPL54* failed to form a complete NLS structural domain with the lack of motif1, and *VcSPL55* failed to form a complete C2HC structural domain without motif2. The others all contain motif1, motif2, and motif3 sequences to form the complete SPL/SBP domain, consistent with the multiple sequence alignment results. Meanwhile, the structural distribution indicated that members of Group1 and Group2 were rich in motifs, while members of Group3 had shorter amino acid sequences and fewer motifs. Some motifs were group-specific, such as motif 6 was only found in Group2, suggesting that they may have unique mechanisms and functions in blueberry.

Within the gene structure, the *VcSPL* gene family was highly variable (Supplementary Table 1 and Fig. S4). Each *VcSPL* gene contains between 2 and 11 exons, with a maximum of 11 exons (*VcSPL1/10/19/24/25*) and a minimum of 1 exon (*VcSPL4/13/16/22/26/38/44*). The complete SPL structural domain (Fig. 3C) was present in all members except for some groups of variants (*VcSPL14*, *VcSPL44,* and *VcSPL54*). And *VcSPL27*, *VcSPL28*, *VcSPL34,* and *VcSPL43* contain an additional intact Ank_2 superfamily structural domain, while *VcSPL15* and *VcSPL36* also contain LRR superfamily and PLN03210 superfamily structural domains. The variety and number of motifs and conserved structural domains differed markedly between the subclasses, which also demonstrates the reliability of our evolutionary classification.

### Chromosomal location and duplication events among VcSPL Genes

**Fig. 4:**
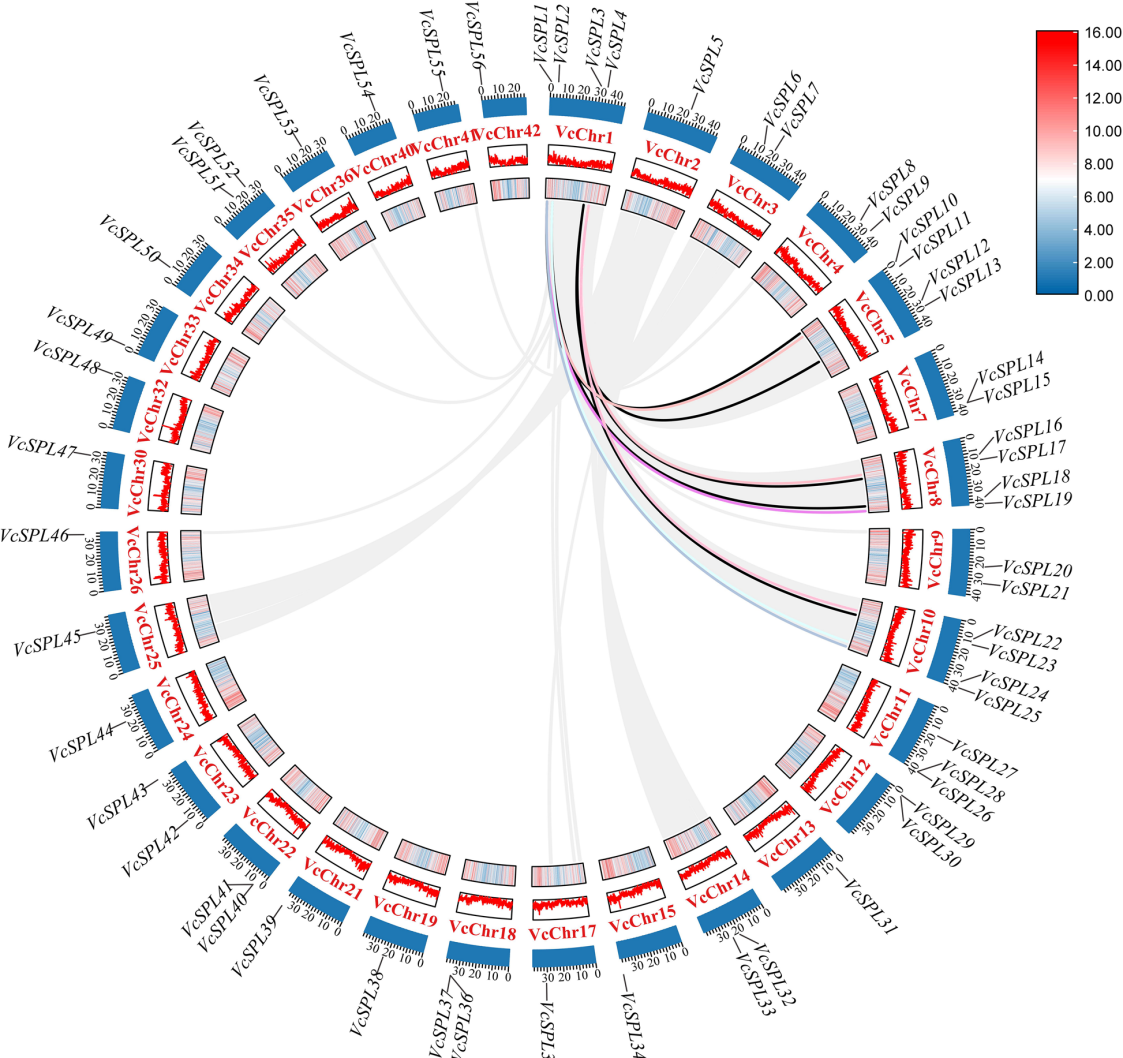
Chromosome distribution and synteny analysis of Vc*SPL* genes. Coloured lines indicate duplication of *VcSPL* genes. The outer blue-filled rectangle represents chromosome length, and the approximate distribution of *VcSPLs* were marked with a short black line on the circle. Gene densities were drawn based on the annotation information of *Vaccinium corymbosum L.* genomes. The two rectangles in the inner circle represent the line pattern and bar pattern of chromosome density respectively, and the legend represents chromosome density

To characterize the expansion patterns of the *VcSPL* genes family, we investigated duplication events in the blueberry genome and compared them with the chromosomal locations of all *VcSPL* genes (Fig. 4). Gene density analysis showed that 56 genes were unevenly distributed on 32 chromosomes, with Chr1,5,10,11 containing a high number of *VcSPL* genes. A total of 11 pairs of segmental duplication genes were found, whereas only two tandem duplications were found in the *VcSPL* gene family (Supplementary Table 4), and all segmental duplications occurred on the first chromosome of *VcSPL1*, *VcSPL2*, *VcSPL3* and *VcSPL4,* and other family members, indicating that segmental duplication played a very important role in *VcSPL* genes expansion and that these four genes also played an important role in the evolution of the *SPL* genes family.

**Fig. 5:**
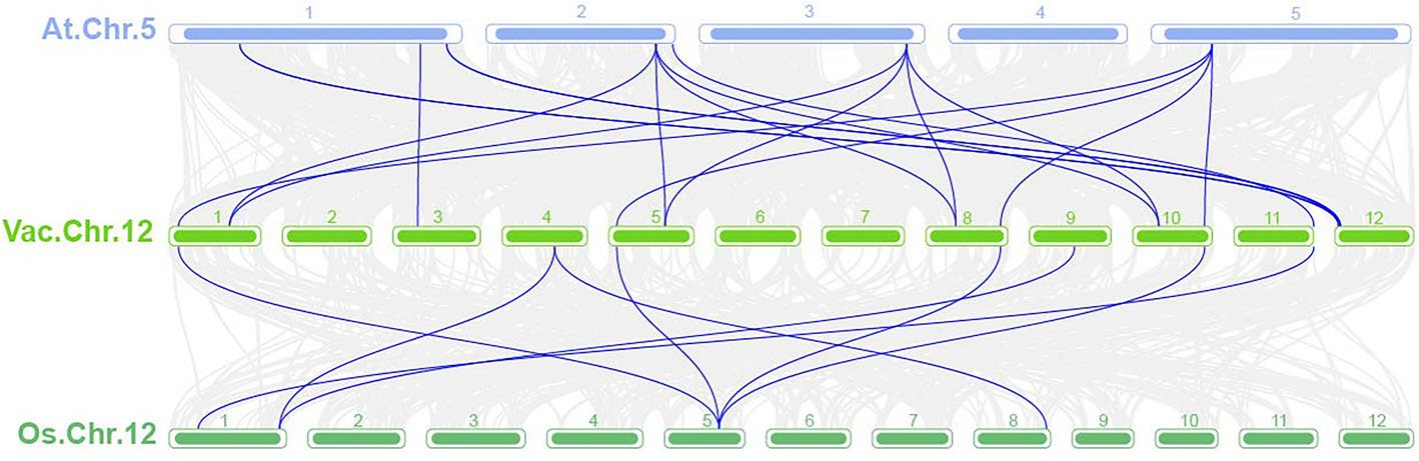
The homologous gene pairs between blueberry and Arabidopsis and blueberry and rice. Grey lines in the background indicate the collinear blocks within blueberry and Arabidopsis and blueberry and rice genomes. Blue lines indicate homologous gene pairs

We constructed a comparative syntenic analysis between blueberry and bilberry genomes to gain more insight into the evolution and differences. The number of 36 homologous pairs has been identified (Fig. S3). We also constructed a comparative syntenic analysis of blueberry and Arabidopsis and rice and identified 16 and 8 pairs of collinear *SPL* genes in Arabidopsis and blueberry, and rice and blueberry (Fig. 5 and Supplementary Table 5), respectively, suggesting that divergence of the common ancestor of rice and dicotyledons occurred before the divergence of blueberry and Arabidopsis. And we found that the collinear genes belonged to the same evolutionary branch (Fig. 2), suggesting that these genes may have similar functions.

### Analysis of the cis-acting element of the VcSPLs promoter

**Fig. 6:**
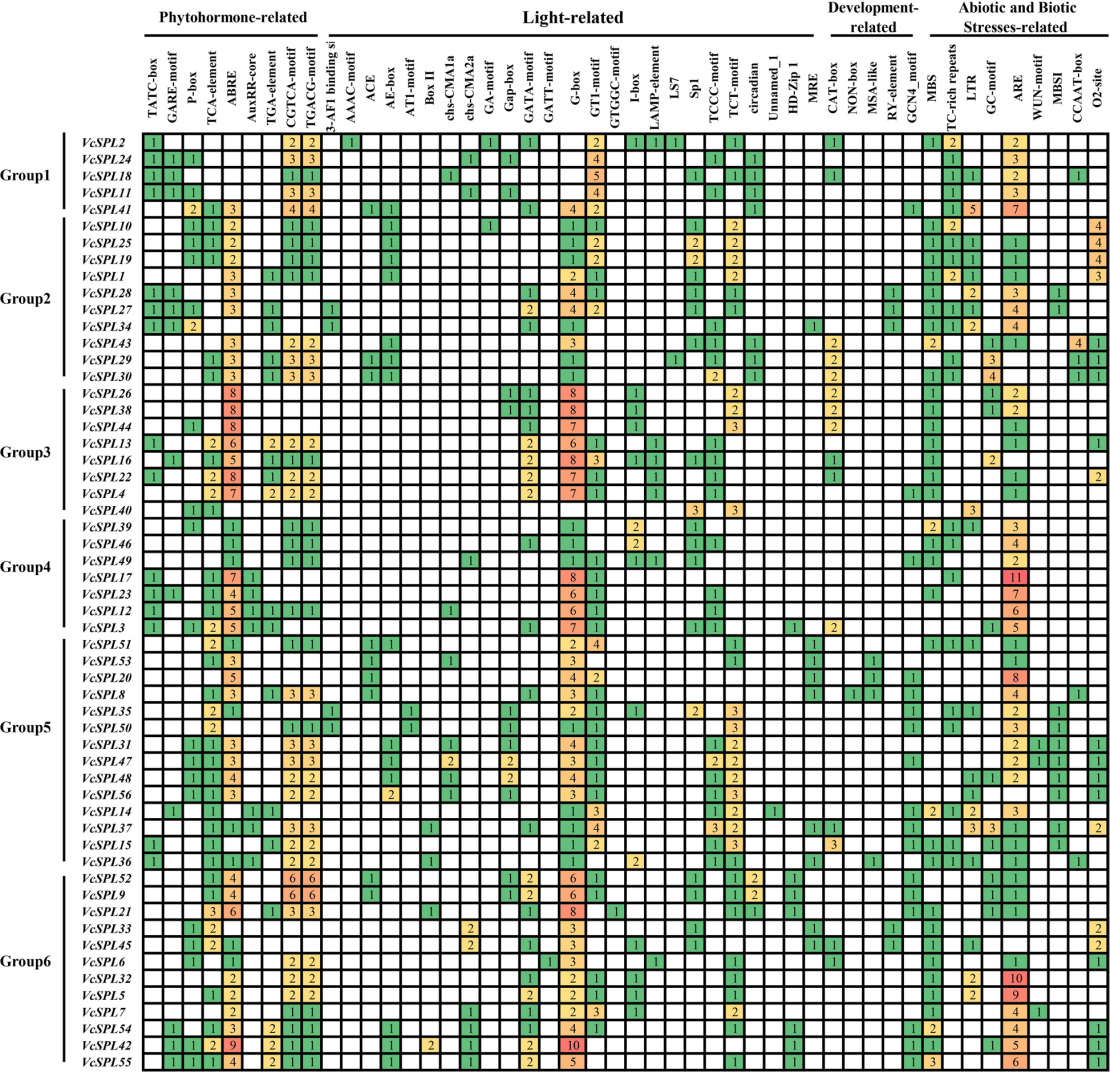
Cis-regulatory elements analysis of the promoter region of *VcSPL* genes. Numbers indicate the number of cis-regulatory elements in the promoter region of the *VcSPL* gene. Based on functional annotation, Vc*SPL* cis-acting elements can be classified into four categories: phytohormones, light response, plant growth and development, and biotic and abiotic stresses

To better understand the response and regulatory mechanisms of Vc*SPL* genes, 2000 bp upstream of the putative translation start codon of genes were selected as cis-acting element sequences. Based on the cis-acting element predictions (Fig. 6 and Supplementary Table 6), it was found that in addition to the basic TATA-box and CAAT-box, cis-acting elements related to phytohormones, light response, plant growth and development, and biotic and abiotic stresses were included. Among the hormone response elements, the abscisic acid responsiveness (ABRE), and the MeJA-responsiveness (TGACG-motif or CGTCA-motif) were the most common of these, suggesting that abscisic acid and MeJA regulated this family of *VcSPL* transcription factors during growth and development. In plant biotic and abiotic stresses, anaerobic induction (ARE) and low-temperature responsiveness (LTR) response elements were more prevalent. Among the plant growth and development-related elements, the meristem expression element (CAT-box) was also detected in large numbers. These results suggested that Vc*SPL* genes play an important role in light response, abscisic acid, MeJA, anaerobic induction, low-temperature responsiveness, and meristem expression.

### Expression pattern of the VcSPLs genes

**Fig. 7:**
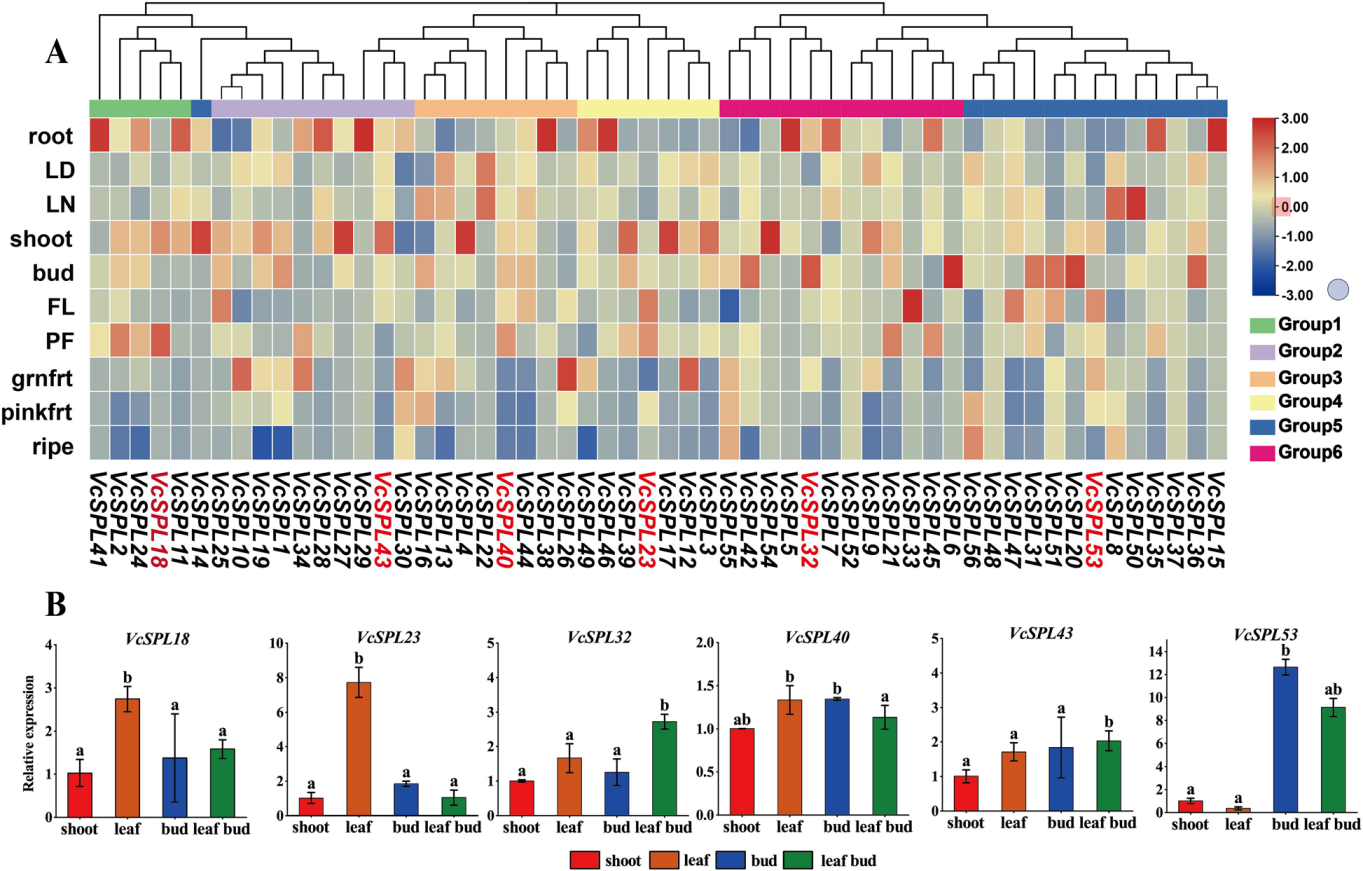
**A** Expression profiles of *VcSPLs* genes in different tissues. The legend represents gene expression, with red representing high expression and blue representing low expression. Clustering from evolutionary branches, Group1-6 represents subgroups of evolutionary branches. LD, Leaf day.LN, leaf, night. FL, Flower at anthesis.PF, Flower post-fertilization.grnfrt, green fruit.pinkfrt, pink fruit.ripe, ripe fruit. **B** Relative expression levels of *VcSPLs* in different tissues of northern highbush blueberry.Lower case letters, when present, indicate significantly different means between different tissue over the same period (using ANOVA, Duncan's test, *p* ≤ 0.05)

Studying the expression pattern of *VcSPLs* in different tissues of blueberry, we found that *VcSPLs* were expressed at higher levels in flower buds, shoots, and roots (Fig. 7A). Considering the materials in our laboratory, genes from each subgroup (*VcSPL18/23/32/40/43/53/54*) were selected for spatiotemporal expression analysis with different tissues (flower buds, leaf buds, shoots, and leaves) of northern highbush blueberry and found that *VcSPLs* were differentially expressed in different tissues (Fig. 7B), with *VcSPL53* being significantly more expressed in flower buds than in other tissues and *VcSPL18/23/40* was significantly higher in leaves than in other tissues, while *VcSPL32/43* was significantly higher in leaf buds than in other tissues. However, their relative expression in different tissues was not consistent with the gene expression profile data, probably due to conditions such as variety, and climatic period.

**Fig. 8:**
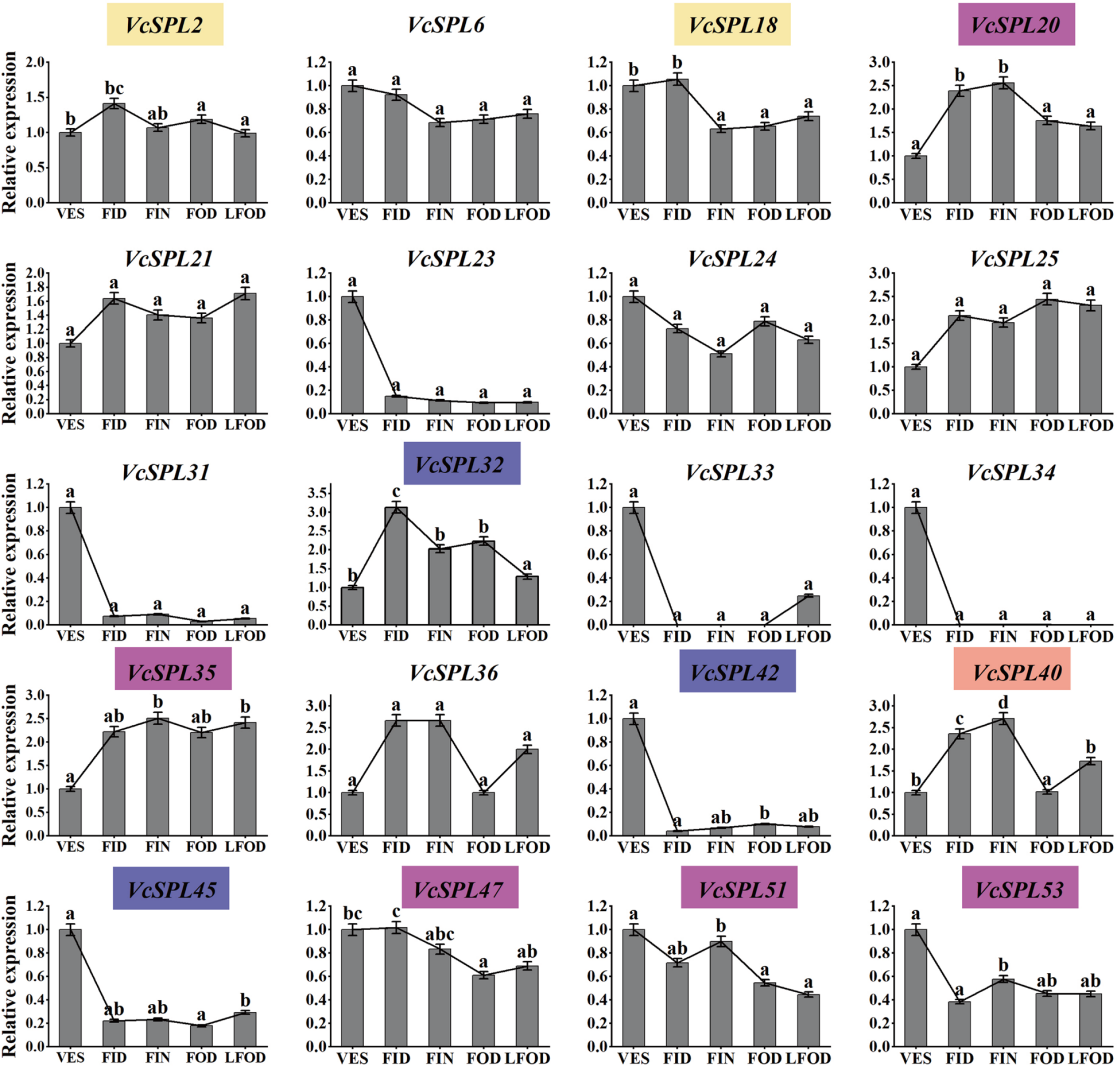
Relative expression of *VcSPL* genes in blueberry bud differentiation at different stages. Lowercase letters, when present, indicate significantly different means between periods for the *VcSPL* gene (using ANOVA, Duncan's test, *P* ≤ 0.05). The coloured ones represent genes that are significantly different at different times and belong to different evolutionary subgroups, yellow for Group1, flesh pink for Group3, rose for Group5, and blue-purple for Group6

However, the different tissue expression profiles only provided changes from bud burst to fruit development. As *VcSPL*s were involved in flower development and *VcSPL*s were expressed at higher levels in buds than in fruits, to further investigate the role of *VcSPL* in floral induction and initiation, we selected genes with high expression in floral tissues for analysis of relative expression during blueberry bud differentiation. *VcSPL* plays different roles in the five periods of blueberry flower bud differentiation (Fig. 8), with *VcSPL2/18/20/32/35/40/42/45/47/51/53* being differentially expressed at different times. According to evolutionary branching, *VcSPL45* was homologous to *AtSPL3*, and this expression was down-regulated during vegetative growth to floral induction, but the difference was not significant. And *VcSPL45* was significantly up-regulated during late floral organ development. *AtSPL13* was part of Group5 in the evolutionary branch, as was *VcSPL20/35/47/51/53*. *VcSPL2/18* was homologous to *AtSPL7* and was significantly down-regulated during floral initiation. *VcSPL20* and *VcSPL35* showed consistent expression trends from vegetative growth to floral initiation, with differences up-regulated, except that *VcSPL20* was significantly down-regulated during flower organ development and *VcSPL35* remained at a higher level. *VcSPL51* and *VcSPL53* showed an overall decreasing trend during blueberry floral bud differentiation. Notably, *VcSPL51* and *VcSPL20* were highly homologous in evolutionary branches, but showed different trends during the blueberry floral transition, indicating that these two genes were not functionally redundant, but functioned separately. *VcSPL32* and *VcSPL42* were homologous to *AtSPL2/10/11*, but the two genes had completely different roles in floral bud differentiation, with *VcSPL32* significantly up-regulated during floral induction but *VcSPL42* significantly down-regulated, and their functional characteristics need to be further investigated, considering their structural domain characteristics and different cis-acting elements.

## Discussion

*SPL* encodes plant-specific transcripts that play important roles in the juvenile-to-adult growth phase transition, flower and fruit development, gibberellin signalling, sporulation, and responses to copper and fungal toxins [54]. Currently, 16, 18, 15 and 18 *SPL* family members have been identified in Arabidopsis [39], rice [41], tobacco [42], and grape [11], respectively, and a total of 56 *VcSPL* gene family members have been identified in tetraploid blueberry in this study. Different plants contain different numbers of *VcSPL* family members, which seems to be caused by functional redundancy or divergence of *SPL* family members during evolution. Notably, 24 *SPL* genes have been previously identified in blueberry [53], indicating that the number of previously identified blueberry *SPL* genes was underestimated, and this experiment further complemented the blueberry *VcSPL* gene family. To investigate the role of *VcSBL* genes in function, we showed the functional diversity of the blueberry *SPL* family in terms of phylogeny, gene structure, chromosomal localization, evolutionary history, and spatiotemporal expression. Firstly, in evolutionary branching and gene structure, genes belonging to the same subclass had the same motif and structural domain characteristics and were clearly distinguished from other subclasses, for example, motif6 was only present in Group2, presumably, this subclass may play a unique mechanism and function in blueberry. The biological functions of genes were often related to their gene expression patterns and structures [53]. In the present study, most members contained SPL structural domains, while Ank_2 superfamily structural domains were found in *VcSPL27*, *VcSPL28*, *VcSPL34,* and *VcSPL43*, and they may be involved in protein interactions [55]. In addition, *VcSPL15* and *VcSPL36* also contain LRR superfamily and PLN03210 superfamily structural domains, which may bind large hydrophobic ligands [56]. Secondly, the *SPL* gene family was suggested to undergo gene duplication in many plants, such as Arabidopsis [39], rice [41], and citrus [9]. Phylogenetic trees indicated that blueberry *SPL* genes were more closely related to bilberry, grape, and Arabidopsis, and more distantly related to rice *SPL* genes. This was consistent with the fact that blueberry, bilberry, grape, and Arabidopsis were dicotyledons. This was also supported by the number of homologous pairs identified in the comparative genomes of blueberry with Arabidopsis and rice, and it was hypothesized that divergence of the common ancestor of rice and dicotyledonous plants occurred before the divergence of blueberry and Arabidopsis. In blueberry, a total of two pairs of tandem duplications were identified and 11 pairs of segmental duplication, presumably segmental duplication playing a very important role in *VcSPL* gene amplification, in agreement with previously [43, 53]. It is clear that blueberry and bilberry show a high degree of clustering at the end of the evolutionary branch of *SPLs*, and 36 homologous pairs were identified, indicating a high degree of convergence in their relationship, consistent with the previous study [43].

Gene expression patterns could provide critical information for studying gene function prediction [57]. In *Prunus mume*, *SPL* genes were expressed at higher levels in flower buds and young fruits [58], and in walnut, eight *JrSBP* genes were expressed at higher levels in female flower buds than in leaf buds during adult floral induction [13]. In the present study, blueberry *VcSPLs* were expressed at higher levels in flower buds, shoots, and roots and lower levels after fruit ripening. The *VcSPLs* genes showed tissue specificity, which implied that the *VcSPL* family may have different functions in blueberries. On the evolutionary branch, *VcSPL18* belonged to Group1, with significantly higher expression in leaves than in other tissues and significantly down-regulated expression during floral initiation. *VcSPL18* was homologous to *AtSPL7,* which in more studies has been involved with the transcription factor *CITF1* in regulating copper uptake and delivery to anthers, thereby affecting fertility [59]. In monocots, down-regulation of *SPL7* and *SPL8* in switchgrass could delay flowering and lead to increased biomass production and sugar release [60]. There were fewer studies on the flower development of *SPL7* in dicotyledons, and the function of *VcSPL18/AtSPL7* needed to be further explored. *VcSPL34/27/28/1/19/25* was in the same branch belonging to Group2 and homologous with *VpSBP5* and *AtSPL1/12/14*. During blueberry bud differentiation, *VcSPL25/34* was not significantly differentially expressed at different stages of blueberry bud differentiation (Fig. 8). Analysis of the cis-acting elements showed that *VcSPL19/25/10* had methyl jasmonate response elements and *VcSPL19/25/10/1* had salicylic acid response elements, and they both also contained low-temperature response elements except for *VcSPL10* (Fig. 6). *AtSPL1/12* has been shown to improve plant heat tolerance and fruit set in Arabidopsis [61], but has not been reported to be involved in regulating flower development. Meanwhile, the *AtSPL1/12/14* genes were involved in the developmental regulation of fumonisin B1 in *Arabidopsis* [62], and the same evolutionary branch, *VpSBP5*, has been shown to prevent powdery mildew in grapes [63]. It has been shown that *VcSPL19* and *VcSPL25* expression was upregulated and *VcSPL10* expression was downregulated 48 h after spraying methyl salicylate [36]. Combined with the results of this experiment, we speculated that Group2 *SPL* genes may be involved in the regulatory pathway of salicylic acid and its function needs to be further investigated. *VcSPL40* belonged to Group3 on the evolutionary branch and was differentially expressed in different periods of blueberry bud differentiation, with expression significantly up-regulated from the vegetative growth phase to floral initiation, significantly down-regulated during floral organ development, and significantly more expressed in leaves than in other tissues (Figs. 7B and 8). In the evolutionary branch, we found that *VcSPL40* had no closer homology to Arabidopsis, but was homologous to *OsSPL7*, a target of miR156f that regulated plant architecture. Overexpression of *OsSPL7* reduced the number of tillers in plants, whereas *OsSPL7* RNAi was shown to increase the number of tillers and reduce plant height [64]. Meanwhile, in the previous study [53], heterologous expression of *VcSPL40* (*VcSBP8b*) was found to exhibit late flowering in *Arabidopsis* and mi156-targeting *VcSPL40*. *SPL* genes targeted by miR156/157 in *Arabidopsis* could directly and positively regulate downstream flowering genes, such as *SOC1*, *LFY*, *AP1,* and *FUL* [18, 19], and GA might also promote flowering by directly binding to SPL transcription factors and activating miR172 and MADS-box genes [20]. Considering that *VcSPL40* showed a gibberellin response element, combined with the expression profile and heterologous expression, we speculated that *VcSPL40* might play a positive role during the floral transition (vegetative growth to floral initiation) and a possible repressive role during floral organ development.

The *AtSPL3* was widely studied for its involvement in floral transition and flowering [21, 23]. In *Antirrhinum majus*, silencing of *AmSBP1* homologous to *AtSPL3/4/5* failed to flower [65]. In the present study, according to the phylogenetic tree, *VcSPL45/33/6* were homologous to *AtSPL3*. In the expression profile, *VcSPL45/33/6* had higher expression during bud, FL, and PF (Fig. 7A), but only *VcSPL45* in blueberry expression was differentially down-regulated during flower bud differentiation (Fig. 8), speculating that the *VcSPL45* gene may play a critical function in the floral induction. The miR156-*SPL* interaction constitutes a cue for endogenous vegetative phase transition and flowering [16], and the vegetative phase change in *Arabidopsis* was regulated by increased expression of *SPL3* as well *SPL4* and *SPL5*, and this increase was the result of decreased miR156 levels [66]. The *BlSPL1*, homologous to *AtSPL3*, played an important role in the change in the vegetative phase [67]. Age-dependent reduction of miR156 led to an increase in *SPL*, which in turn activated the expression of miR172, MADS-box genes, and *LFY* genes [20]. Currently, miR156/157 has been shown to target *VcSPL45* (*VcSBP6a*) [31], but the regulatory pathway of miR156-*VcSPL45* with downstream flowering genes (*LFY, MADX-box*) in blueberry flower formation needs to be further explored.

*AtSPL9/15* was involved in juvenile-to-adult vegetative transformation [15, 16] and might play a functionally redundant role. In Petunia, *PhSPL9a* and *PHSPL9c* showed high transcript levels in the inflorescence, but *PhSPL9b* was expressed in all tissues [57]. In Walnut, *JrSBP19*, *JrSBP20,* and *JrSBP24* were homologous to *AtSPL9/15*, and during floral induction, *JrSBP19* and *JrSBP20* were highly expressed in leaf buds, but *JrSBP24* was highly expressed in flower buds [13]. In this study, *VcSPL39/46/49/17/23/12/3* were homologous to *AtSPL9/15*, where the *VcSPL* expression profile showed that *VcSPL23* was highly expressed during PF and FL (Fig. 7A). RT-qPCR results showed that *VcSPL23* was significantly more expressed in leaves but not significantly expressed during blueberry bud differentiation (Figs. 7B and 8). *AtSPL9* was associated with leaf growth rate and final size [17], and we speculated that *VcSPL23* may function mainly in flower bursts and leaves. Tomato *SlSPL13* positively regulated the expression of the tomato inflorescence-related gene *SINGLE FLOWER TRUSS (SFT)* by binding directly to its promoter region, thereby controlling inflorescence development [68]. In *Medicago sativa*, delayed flowering was observed in *SPL13*-silenced plants [69]. *AtSPL13* was part of Group5 in the evolutionary branch, as was *VcSPL20/35/47/51/53*. However, *VcSPL35* has higher homology with *OtSPL16* in the evolutionary branch, and it has been reported that *OsSPL16* positively conditions cell proliferation, and *OsSPL16* missense mutations generate new alleles that affect plant height and inflorescence development [70], specifically showing dwarfism and shortened inflorescence length [71]. The expression of *VcSPL35* was differentially up-regulated during blueberry flower bud differentiation, and it was hypothesized that *VcSPL35* played an active role in promoting blueberry flower bud differentiation. *VcSPL53* was significantly up-regulated during flower initiation, and was significantly higher in blueberry flower buds than in other tissues (Figs. 7B and 8). Meanwhile, gene structural analysis showed that *VcSPL53* had salicylic acid response elements. Under long daylight, salicylic acid (SA) can induce flowering in Wolffia microscopic of short daylight plant [72], salicylic acid treatment can promote more flowering in citrus [73], and salicylic acid (SA) accumulation can inhibit the development of stamen abscission in tung tree [74]. Flower bud differentiation and flowering are two distinct stages in most woody plants [75, 76], and it can also be hypothesized that *VcSPL53* is involved in floral initiation and flowering in blueberry. In summary, the blueberry *VcSPL* gene family has a significant role in blueberry plant growth, leaf development, flowering regulation, and abiotic stress, especially in the process of floral initiation and induction.

## Conclusion

In this study, we performed a genome-wide identification and analysis of *VcSPL* in the blueberry. A total of 56 *VcSPL* gene families were identified, grouped into six subfamilies on the phylogenetic tree, and the results were further supported by gene structural domain, and motif analysis. 56 *VcSPL* genes were localized to the nucleus except for *VcSPL36*, which was localized in both the cytoplasm and the nucleus. In addition, the analysis of evolutionary origins and cis-acting elements provided further insight into the function of the *VcSPL* gene family, and segmental duplications may play a key role in *VcSPL* gene amplification. Expression profiles and RT-qPCR results showed that *VcSPL* genes were not only spatially and temporally specific, but also could play important roles in blueberry plant growth, leaf development, flower development, and abiotic stresses, with some genes acting synergistically. Among them, *VcSPL40*, *VcSPL35*, *VcSPL45,* and *VcSPL53* may have critical roles in the floral transition of blueberries. This study will provide a theoretical basis for studying the blueberry flower and fruit development and improving agronomic traits.

## Main conclusion

We identified 56 *SPL* genes by bioinformatic analyses. *VcSPL40*, *35*, *45*, and *53* play a role in the blueberry floral transition phase. 

## Supplementary Information

**Additional file 1:** **Figure S1.** Multiple sequence alignment of* SPL* protein in blueberry. **Figure S2.** Analysis of conserved motifs of *SPL* protein in blueberry. **Figure S3.** The homologous gene pairs between blueberry and bilberry. **Figure S4.** Gene structure of the blueberry *SPL* gene. The left image represents evolutionary branching, and the right represents the visualization of gene structure, green and yellow represent CDS and UTR respectively.

**Additional file 2:** **Table S1.** Basic information of the SPL gene family in blueberry. **Table S2.** Gene names and locus information for the SPL proteins in Bilberry. **Table S3.** Correspondence between the previously identified SPL gene family and the SPL genes identified in this study. **Table S4.** Tandem duplicationsand segmental of VcSPL gene pairs in Vaccinium corymbosum. **Table S5.** Synteny analysis between Vaccinium corymbosum and Arabidopsis thaliana/Oryza sativa. **Table S6.** Motif and number of cis-acting elements corresponding to the promoter region of the blueberry VcSPL gene. **Table S7.** Primer sequences of VcSPL for RT-qPCR.

## Data Availability

The experimental materials were collected from the blueberry base of Junxing Guangda Specialized Cooperative of Agricultural Products and obtained the permission of the cooperative. The blueberry (*Vaccinium corymbosum*) genome data has been published [36], and GigaScience fully supports the National Academy of Sciences recommendations for data sharing. All data generated or analysed during this study are included in this published article and its supplementary information files. The datasets generated and/or analysed during the current study are available in the Blueberry Database repository, https://www.vaccinium.org/.
