# Supplementary material for: Molecular characterization of SPL gene family during flower morphogenesis and regulation in blueberry
Source: BMC Plant Biol. 2023 Jan 18;23:40. doi: 10.1186/s12870-023-04044-x (PMC9847132; doi:10.1186/s12870-023-04044-x)

Figure S1. Multiple sequence alignment of *SPL* protein in blueberry.


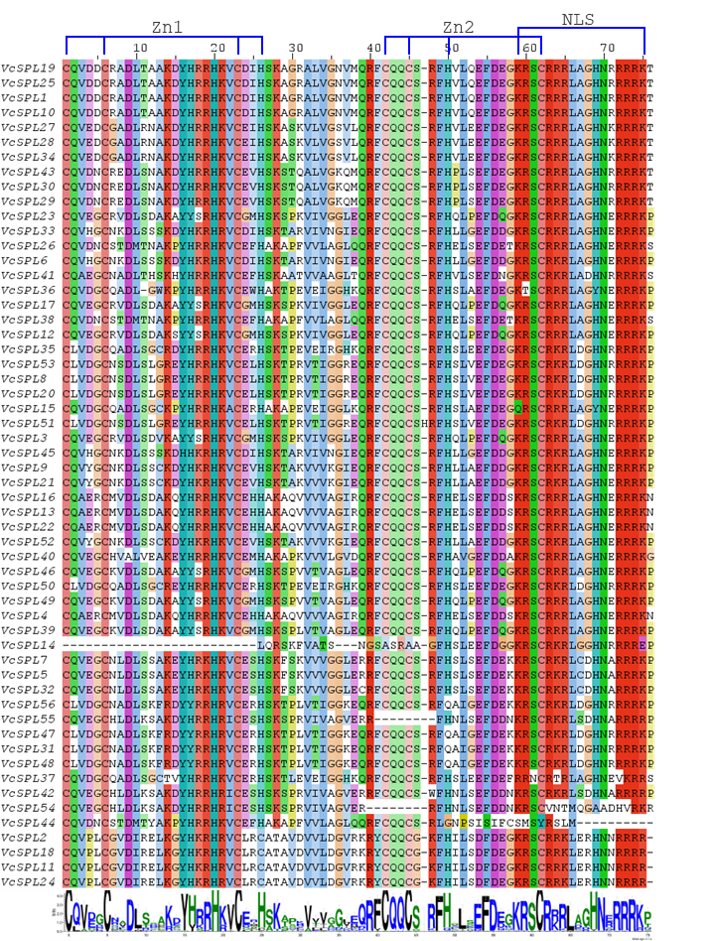


Figure S2. Analysis of conserved motifs of *SPL* protein in blueberry.


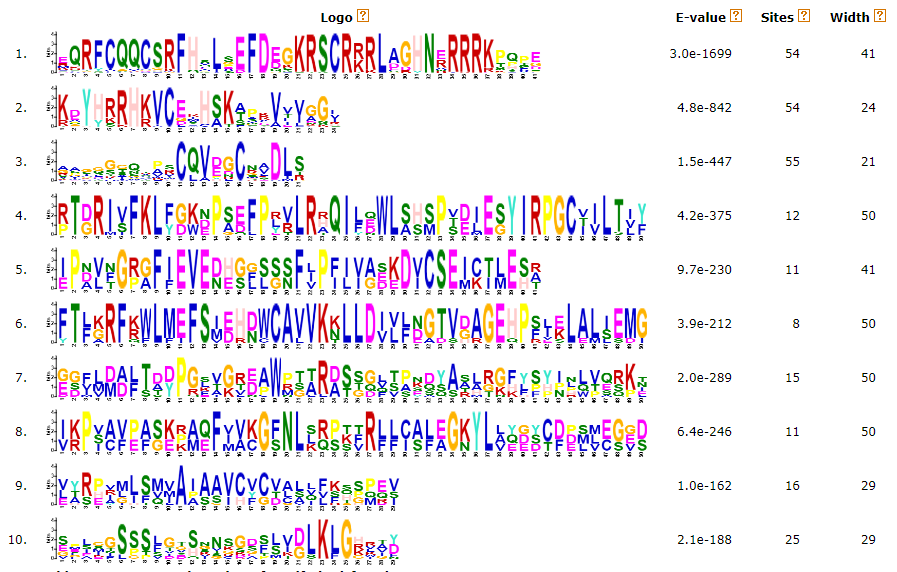


Figure S3. The homologous gene pairs between blueberry and bilberry.


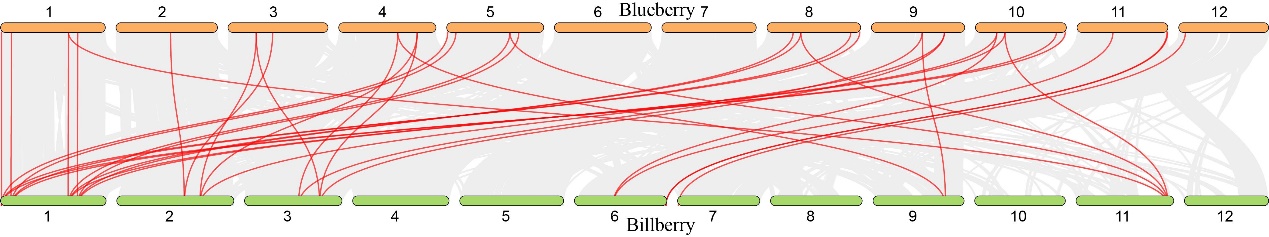


Figure S4. Gene structure of the blueberry *SPL* gene. The left image represents evolutionary branching, and the right represents the visualization of gene structure, green and yellow represent CDS and UTR respectively


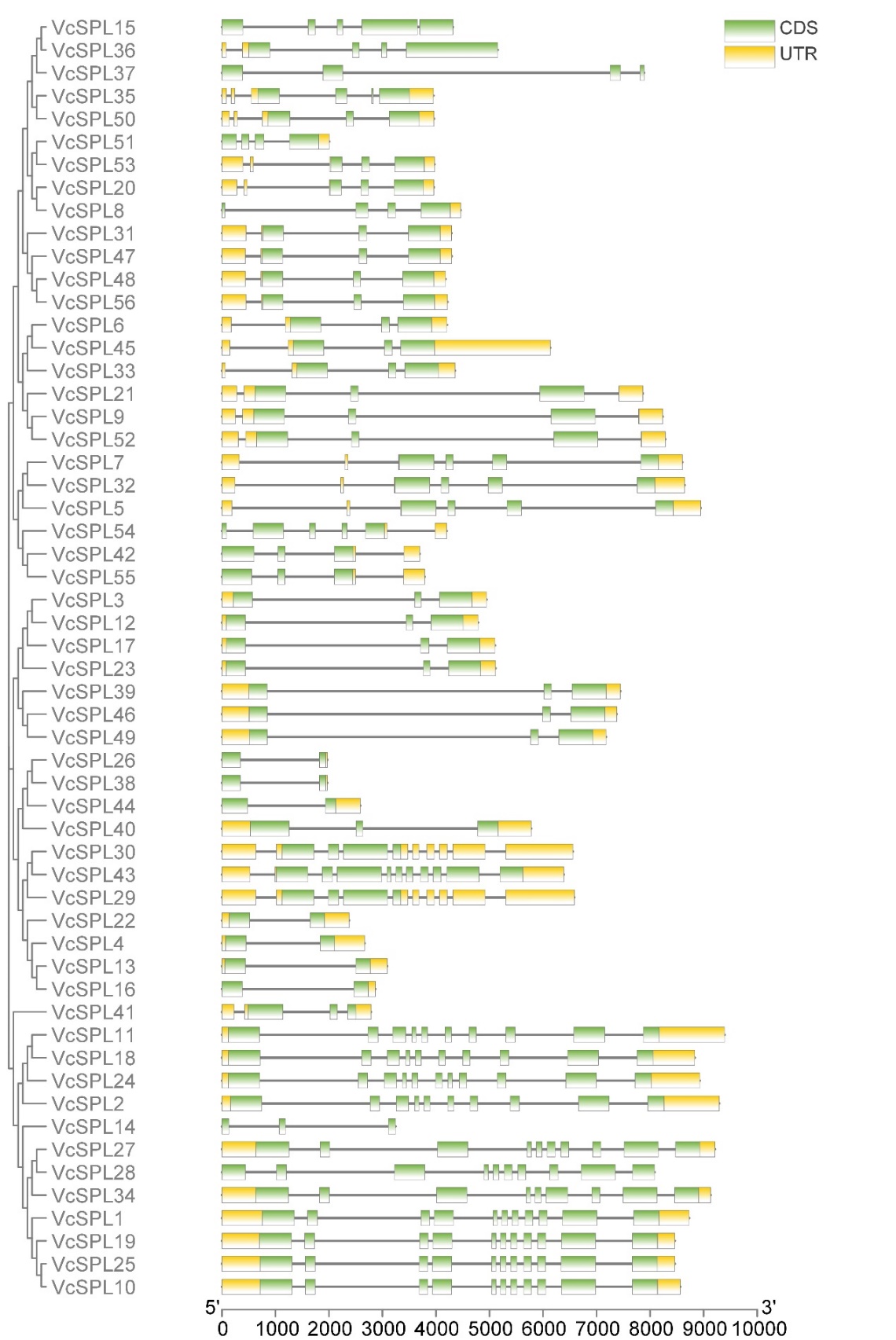

Supplement: Supplementary file 1 — Additional file 1: Figure S1. Multiple sequence alignment of SPL protein in blueberry. Figure S2. Analysis of conserved motifs of SPL protein in blueberry. Figure S3. The homologous gene pairs between blueberry and bilberry. Figure S4. Gene structure of the blueberry SPL gene. The left image represents evolutionary branching, and the right represents the visualization of gene structure, green and yellow represent CDS and UTR respectively. [file 12870_2023_4044_MOESM1_ESM.docx]
